# Osmotic and Heat Stress Effects on Segmentation

**DOI:** 10.1371/journal.pone.0168335

**Published:** 2016-12-22

**Authors:** Julian Weiss, Stephen H. Devoto

**Affiliations:** Department of Biology, Wesleyan University, Middletown, Connecticut, United States of America; Texas A&M University, UNITED STATES

## Abstract

During vertebrate embryonic development, early skin, muscle, and bone progenitor populations organize into segments known as somites. Defects in this conserved process of segmentation lead to skeletal and muscular deformities, such as congenital scoliosis, a curvature of the spine caused by vertebral defects. Environmental stresses such as hypoxia or heat shock produce segmentation defects, and significantly increase the penetrance and severity of vertebral defects in genetically susceptible individuals. Here we show that a brief exposure to a high osmolarity solution causes reproducible segmentation defects in developing zebrafish (*Danio rerio*) embryos. Both osmotic shock and heat shock produce border defects in a dose-dependent manner, with an increase in both frequency and severity of defects. We also show that osmotic treatment has a delayed effect on somite development, similar to that observed in heat shocked embryos. Our results establish osmotic shock as an alternate experimental model for stress, affecting segmentation in a manner comparable to other known environmental stressors. The similar effects of these two distinct environmental stressors support a model in which a variety of cellular stresses act through a related response pathway that leads to disturbances in the segmentation process.

## Introduction

All vertebrates are segmented. These segments arise during early embryonic development, when the paraxial mesoderm becomes subdivided into somites. The molecular and cellular mechanisms of somite development are generally conserved between all vertebrates that have been examined [1]. Unsegmented presomitic mesoderm (PSM) expresses a set of genes—the segmentation clock—at levels that oscillate with the same temporal periodicity as segmentation. The PSM is simultaneously exposed to overlapping gradients (wavefronts) of FGF, WNT, and retinoic acid signaling. Near the anterior end of the PSM, a gene regulatory network involving the *tbx6*, *mesp*, and *ripply* genes is activated. This network is part of a reading head that translates the information from the clock genes and the wavefront into the morphogenetic process of segmentation.

Defects in this segmentation process can lead to skeletal and muscular deformities including several clinical conditions [2]. These range from severe vertebral defects, common in disorders such as spondylocostal dysostosis [3], to milder deformities, such as congenital scoliosis (CS) [4]. Mutations in human genes integral to the segmentation process, including the clock genes *HES7*, *DLL3*, and *LFNG*, as well as the reading head gene *MESP2*, underlie many of the more severe vertebral defects in humans [5]. Though such recessive monogenic traits are often the causative factor of severe vertebral disorders in humans, the underpinnings of more subtle vertebral anomalies like CS are not understood as well. CS is associated with mutations in genes such as *TBX6*, though genetic factors generally have a low penetrance and as such are unlikely to be the sole contributing factor to CS [6]. Environmental embryonic conditions may also contribute to many cases of CS, likely increasing the severity and penetrance of the defects caused by genetic mutations [7]. This environmental influence is not completely unexpected, as a variety of environmental stressors can disrupt segmentation and vertebrae development in model organisms independent of genetic mutations [8].

One of these stressors, a brief heat shock during the segmentation period of embryogenesis, leads to somite defects in zebrafish [9], chick [10], frog [11], and salmon [12]. Hypoxic treatment also induces segmentation disturbances in mouse embryos [7]. These effects are reproducible and occur in a similar location relative to treatment across species and even environmental stresses, possibly suggesting a common mechanism by which these stressors cause segmentation defects. Furthermore, both hypoxia and heat shock cause stress on the endoplasmic reticulum (ER), an organelle involved in protein folding, protein maturation, and the secretory pathway [13]. ER stress occurs when misfolded proteins accumulate in the ER. Such ER dysfunction results in activation of the unfolded protein response (UPR), which is meant to clear the buildup of misfolded proteins, thereby restoring cellular homeostasis and proteostasis [14]. This is accomplished by upregulation of both protein chaperones and proteases, and decreasing protein production. The common mechanism by which these stressors cause segmentation defects may therefore involve the induction of ER stress and activation of the UPR.

Hypertonic stress causes an intracellular ionic imbalance, which can trigger protein denaturation and aggregation [15,16], thus causing ER stress. Osmotic stress, hypoxia, and heat stress have all been shown to cause ER stress and activate the cellular stress response through several pathways [17,18]. However, whether osmotic stress affects segmentation has not yet been examined.

Here, we describe experimental conditions which result in reproducible osmotic stress-induced defects in somite border formation in zebrafish embryos. We show that both osmotic shock and heat shock produce border defects in a dose-dependent manner, with an increase in both frequency and severity of defects. We also show that osmotic treatment has a delayed effect, similar to that observed in heat shocked embryos. That is, hyperosmolar treated embryos exhibit defects in somite borders that formed following, rather than during, treatment. The similar effects of these two distinct environmental stressors supports a model in which a variety of cellular stresses act through a related response pathway, conserved across species, that leads to disturbances in the segmentation process.

## Materials and Methods

### Animals

All animal experiments were undertaken according to protocols approved by the Wesleyan University Animal Care and Use Committee, assurance number A3956-01.

### Zebrafish, osmotic shocks, heat shocks

All experiments were conducted using wild-type zebrafish (*Danio rerio*) from the Wesleyan University strain of wild-type fish. Embryos were cared for using standard procedures [19].

Osmotic shocks were conducted on embryos at the indicated stages by transferring them in small mesh-bottom wells to a high osmolarity solution for 45 minutes. These solutions consisted of standard zebrafish embryo medium containing additional sodium chloride or dextrose, which were added to increase the osmolarity of the solution. The embryos were returned to normal embryo medium following treatment to continue development.

Embryos were heat shocked in a similar manner, but were transferred to embryo medium pre-heated to the indicated temperatures for 45 minutes, then returned to standard temperature (28.5°C) to continue development.

### Scoring segmentation defects

Following heat or osmotic shock, embryos were maintained in embryo medium at 28.5°C until 72hpf. Embryos were then anesthetized with a tricaine mesylate solution and viewed using polarized light through a Leica microscope. The affected somite borders were recorded relative to the time of treatment according to the standard nomenclature (i.e., the most recently formed somite and somite border at the time of shock are SI and B0, respectively) [20]. The severity of somite border defects was scored using the following criteria for each embryo.

No defects:

Segment borders were regularly spaced, and extended in an uninterrupted manner from dorsal to ventral, parallel to each other.

Mild Defects:

One or more segment borders was not parallel to the others, or was irregularly spaced, thereby creating one or more large, or small, irregularly shaped myotome. One or more segment borders with approximately 20% or less of the border missing.

Moderate defects:

In addition to those defects seen in “mild”, one or more borders are missing up to 50% of the border or are more irregularly shaped than those defects classified as mild.

Severe defects:

In addition to those defects seen in “moderate”, one or more borders are missing more than 50% of the border or are otherwise severely misshapen.

Periodic defects:

Multiple defects of any sort that occurred on at least one side of the embryo and were separated by at least two normally formed somites.

## Results

### Osmotic stress and heat stress produce border defects in a dose-dependent manner

**Fig 1:**
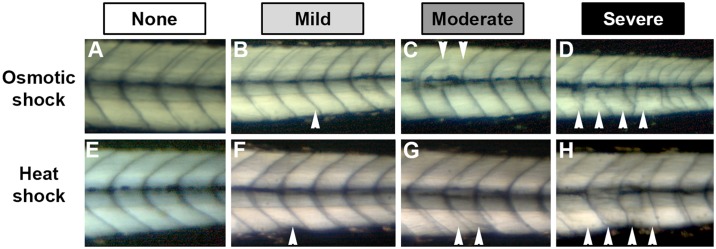
Osmotic and heat shock lead to variable segment border defects. Embryos were shocked at the 9 somite stage with a 840 mOsm solution (A-D) or 37°C embryo medium (E-H) for 45 minutes and allowed to develop until approximately 72 hpf. Arrowheads point to visible somite border defects. (A,E) Embryos exhibiting no defects. (B) The ventral portion of a somite border is curved. (C) The dorsal half of a somite border failed to form. (D) Multiple irregularly-formed somite boundaries. (F) A somite border is curved and the somite is narrow. (G) The ventral portion of two consecutive borders failed to reach the end of the somite. (H) Multiple irregularly-formed somite borders.

Zebrafish embryos undergo normal development at osmolarities between approximately 10 mOsm to 200 mOsm [21]. We find that exposing embryos to a hypertonic environment with an osmolarity greater than approximately 750 mOsm for a period of 45 minutes during somitogenesis induced disturbances in the developing somites (Fig 1). These effects were not solely due to the effect of saline, as we observed border defects in embryos exposed to high osmolarity solutions using dextrose instead of sodium chloride (data not shown). Following osmotic shock, embryos exhibited a wide array of defects from mild to severe (see segmentation defect scoring system in Methods; Fig 1B–1D).

**Fig 2:**
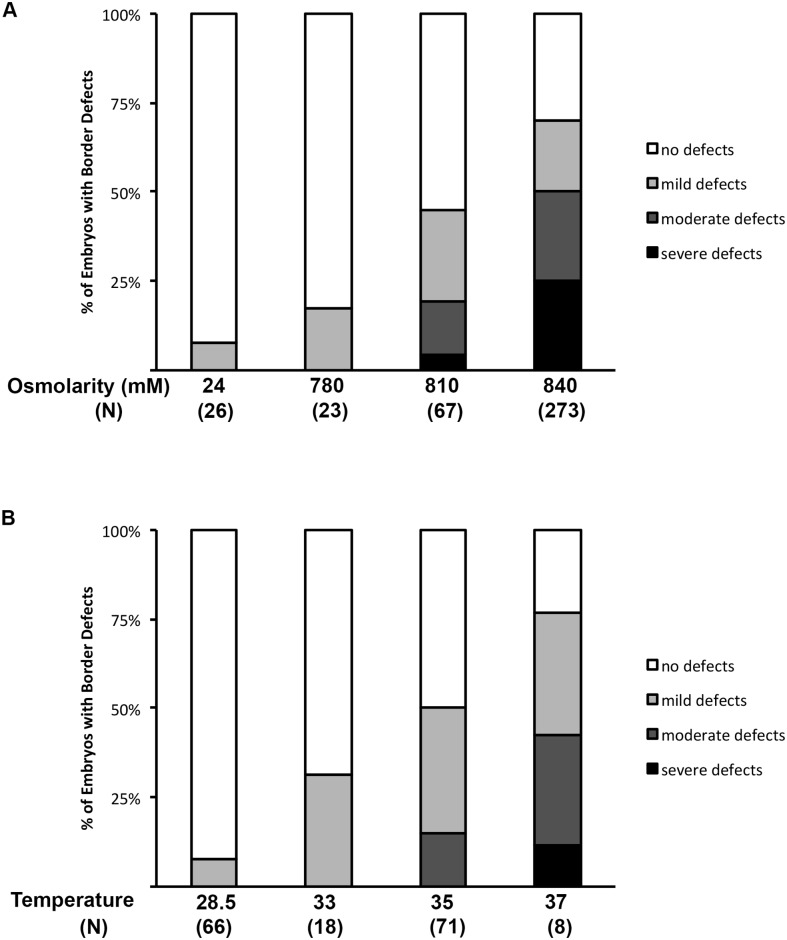
Osmotic and heat shock produce somite border defects in a dose-dependent manner. (A) Osmotic shock dose response curve. (B) Heat shock dose response curve. Graphs represent the percentage of embryos in each category. Severity of somite border defects was determined using polarized light microscopy.

As we increased the osmolarity, the frequency and severity of somite border defects increased (Fig 2A). Notably, relatively small increases in osmolarity sharply increased the rate and severity of defects. Osmotic treatments done at 810 mOsm produced defects at greater than twice the rate of a 780 mOsm treatment. Furthermore, we observed a significant increase in the frequency of moderate and severe defects at 840 mOsm, as compared to the frequency at 780 mOsm and 810 mOsm.

We find that the hyperosmolar treatment produced segmentation defects that were not qualitatively identical to those commonly observed in heat shocked embryos. While heat shock typically produces somite border defects in one to three consecutive somites (Fig 1F–1H), osmotic shock frequently lead to border defects in six or more consecutive somites (e.g., Fig 1D). Such large domains of border disturbances were also observed in embryos exposed to a hyperosmotic dextrose solution (data not shown). The types of border defects observed also varied between the two environmental stresses. While both treatments lead to mild and moderate defects that are similar in appearance, osmotic shocked embryos with severe defects are easily distinguishable from heat shocked embryos with severe defects (Fig 1D and 1H). Severe border defects in hyperosmolar treated embryos exhibit a disorganized appearance, as the affected borders are often misshapen and occasionally even intersect with an adjacent border. In contrast, the severe defects we observed in heat shocked embryos tended to be those in which large portions of the border were missing, as opposed to defects characterized by borders with a highly irregular shape.

Quantitatively, however, osmotic and heat shock were comparable. At increasing heat shock temperatures, the frequency and severity of boundary disruptions increased, matching the trend observed for osmotic shock treatments (Fig 2). Thus both heat and osmotic stresses affect the somitogenesis process of the zebrafish embryo in a dose-dependent manner. These data are consistent with the dose response curve obtained by examining vertebral defects upon hypoxic treatment of mouse embryos [7]. As with the effects of hypoxia on vertebrae in the mouse embryo, a greater stress causes a greater number of defects in the zebrafish embryo.

### Hyperosmolar treated embryos exhibit defects in a particular location

In zebrafish, PSM cells become determined to segmentation about two hours prior to the formation of a border [22]. As somites form every 30 minutes, the segment determination front is about four segmentation cycles posterior to the last formed somite. In heat shocked embryos, the somite present at the time of heat shock and the next several somites develop normally [9]. After these unaffected somites, a border defect is observed. This developmental delay in the effect of heat shock on segmentation is consistent with data indicating that segment determination occurs 4–6 segment cycles prior to the formation of physically separated segments (somites).

**Fig 3:**
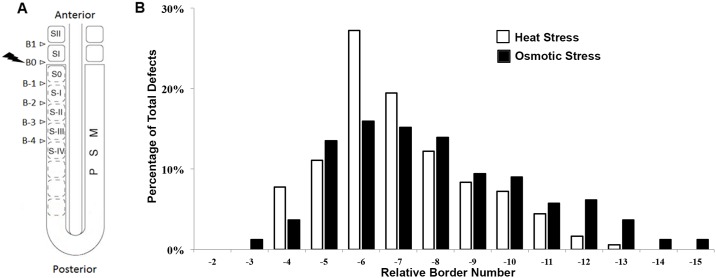
Osmotic and heat shock result in defects centered in the same area. (A) Visualization of somitogenesis to illustrate formed and developing somites and relative border numbers. Lightning bolt indicates where shock begins. (B) Proportion of defects in hyperosmolar shocked (black) and heat shocked (white) embryos, shown relative to the stage at which the osmotic or heat treatment began.

Both heat shock and osmotic shock produced defects in somite borders formed after treatment, rather than affecting the border developing at the time of treatment (Fig 3B). For both treatments the defects were centered, on average, at border B-6 (27% of heat shock defects and 16% of osmotic shock defects). Importantly, the stress did not affect somites forming earlier, anterior to this region, or later, posterior to this region. This suggests that osmotic shock affects the presomitic mesoderm that is in the segmentation determination front at the time of the shock. The clock components, the wavefront components, and the reading head components form interconnecting gene regulatory networks; each gene in these networks is a candidate to be the direct target of an environmental stressor. In mice, hypoxic stress is exacerbated by partial loss of function alleles in clock components as well as in reading head components, supporting a role for these genes in the response to hypoxic stress.

Notably, however, the distribution of somite border defects skewed posterior in hyperosmolar shocked compared to heat shocked embryos. While both treatments caused defects centered at border B-6, osmotic treatment caused border defects in B-9 or a more posterior border at a significantly greater frequency than in heat shocked embryos. We suspect this discrepancy between osmotic and heat stress effects likely results from a difference in the nature of the stressors. While temperature has an immediate effect (i.e., the embryonic tissue is raised to the temperature of its environment), an increase in osmolarity takes time both to enter the cells and to leave the cells, resulting in a longer lasting stress and a consequent posterior shift in the incidence of defects.

## Discussion

The phenomenon that heat shock causes segmentation disturbances has been well documented, and more recent studies have shown that hypoxic stress induces such defects as well in some organisms. Here we have identified another environmental stress, osmotic stress, that causes segmentation defects in zebrafish. The disruptive effect on segmentation is dose dependent, similar to hypoxia and heat shock. Furthermore, osmotic stress causes defects located in a particular location relative to the time of treatment. This peak of defects occurs in a similar relative location as heat shock. The delayed response between treatment and defect indicates that osmotic shock affects the molecular machinery involved late in the segmentation process.

Candidates for targets of these stressors are proteins required for the spatial gradients of growth factors (the wavefront), and proteins required for the “reading head” that translates temporally cycling gene expression (the clock), in conjunction with spatial gradients to specify segments. Proteins expressed in the area of the reading head include Tbx6, Ripply1, and Mesp-b [23]. In the mouse embryo, hypoxic exposure results in the downregulation of the gradient proteins FGF and Wnt [7]. Each of these is integral to the segmentation process in zebrafish, and thus may contribute to the morphological defects observed in heat shocked and osmotic shocked embryos. Future experiments measuring RNA and protein expression of these molecules following environmental stress may help answer this outstanding question.

## Supporting Information

S1 Table Percentage of embryos with defects. (XLSX)

S2 Table Relative location of defects in treated embryos. (XLSX)
